# Exosome‐delivered miR‐410‐3p reverses epithelial‐mesenchymal transition, migration and invasion of trophoblasts in spontaneous abortion

**DOI:** 10.1111/jcmm.18097

**Published:** 2024-01-02

**Authors:** Zhen‐yue Chen, Zhen Li, Yun Zong, Bo Xia, Song‐ping Luo, Gao‐pi Deng, Jie Gao

**Affiliations:** ^1^ The First Clinical Medical College of Guangzhou University of Chinese Medicine Guangzhou China; ^2^ Lingnan Medical Research Center of Guangzhou University of Chinese Medicine Guangzhou China; ^3^ The Second Clinical College of Guangzhou University of Chinese Medicine The Second Affiliated Hospital of Guangzhou University of Chinese Medicine Guangzhou China; ^4^ Department of Gynecology First Affifiliated Hospital of Guangzhou University of Chinese Medicine Guangzhou China

**Keywords:** EMT, miR‐410‐3p, p38 MAPK signalling pathway, serum exosomes, spontaneous abortion, TRAF6

## Abstract

Current studies have indicated that insufficient trophoblast epithelial‐mesenchymal transition (EMT), migration and invasion are crucial for spontaneous abortion (SA) occurrence and development. Exosomal miRNAs play significant roles in embryonic development and cellular communication. Hereon, we explored the roles of serum exosomes derived from SA patients on trophoblast EMT, migration and invasion. Exosomes were isolated from normal control (NC) patients with abortion for unplanned pregnancy and SA patients, then characterized by transmission electron microscopy (TEM), nanoparticle tracking analysis (NTA) and western blotting. Exosomal miRNA profiles were identified by miRNA sequencing. The effects of serum exosomes on trophoblast migration and invasion were detected by scratch wound healing and transwell assays, and other potential mechanisms were revealed by quantitative real‐time PCR (RT‐PCR), western blotting and dual‐luciferase reporter assay. Finally, animal experiments were used to explore the effects of exosomal miR‐410‐3p on embryo absorption in mice. The serum exosomes from SA patients inhibited trophoblast EMT and reduced their migration and invasion ability in vitro. The miRNA sequencing showed that miR‐410‐3p was upregulated in SA serum exosomes. The functional experiments showed that SA serum exosomes restrained trophoblast EMT, migration and invasion by releasing miR‐410‐3p. Mechanistically, SA serum exosomal miR‐410‐3p inhibited trophoblast cell EMT, migration and invasion by targeting TNF receptor‐associated factor 6 (TRAF6) at the post‐transcriptional level. Besides, SA serum exosomal miR‐410‐3p inhibited the p38 MAPK signalling pathway by targeting TRAF6 in trophoblasts. Moreover, milk exosomes loaded with miR‐410‐3p mimic reached the maternal‐fetal interface and aggravated embryo absorption in female mice. Clinically, miR‐410‐3p and TRAF6 expression were abnormal and negatively correlated in the placental villi of SA patients. Our findings indicated that exosome‐derived miR‐410‐3p plays an important role between SA serum and trophoblasts in intercellular communication, suggesting a novel mechanism by which serum exosomal miRNA regulates trophoblasts in SA patients.

## 1 INTRODUCTION

Spontaneous abortion (SA) comprises a spontaneous pregnancy termination before the foetus is viable, including all miscarriages from conception to the 24th fetation week.^1^, ^2^ Approximately 23 million SAs occur worldwide each year, representing 44 miscarriages per minute.^3^ Despite 15% of clinically diagnosed miscarriages, the total reproductive loss is close to 50%.^4^, ^5^ SA is a sophisticated pathological process involving multiple factors at the maternal‐fetal interface, jeopardizing women's reproductive health.^6^, ^7^, ^8^ During the human placenta's early development, cytotrophoblasts (CTBs) in the villi proliferate and differentiate into syncytiotrophoblasts (STBs) and extravillous trophoblasts (EVTs).^9^ EVTs are important placenta components and exert critical roles in embryo implantation and gestation.^10^ In 1996, the epithelial‐mesenchymal transition (EMT) was proposed to be involved in CTB differentiation to EVT.^11^ The EMT is characterized by epithelial phenotype loss and mesenchymal phenotype gain and regulates EVT migration and invasion.^12^ Therefore, investigating the underlying mechanisms of the EMT in EVTs is essential to better understand SA etiopathogenesis.

Furthermore, microRNAs (miRNAs) are endogenous, non‐coding, 22‐nucleotide, single‐stranded protein RNAs, widely present in eukaryotic cells.^13^ The 3′‐UTR is primarily targeted in mRNA regulation.^14^ Increasing evidence has shown that abnormal miRNA expression is linked to reproductive disorders such as RSA. Mitogen‐activated protein kinase (MAPK) pathways are crucial response regulators for many extracellular stimuli. The p38 MAPK signalling pathway, conserved from yeast to mammals, has four members: p38a (MAPK14), p38b (MAPK11), p38d (MAPK13) and p38g (MAPK12).^15^ Although p38 kinase is primarily associated with stress and inflammation, it participates in cell proliferation, cell cycle regulation and differentiation.^16^ Additionally, p38 MAPK can maintain pregnancy and promote labour, but its inappropriate activation can lead to adverse pregnancy outcomes.^17^ Additionally, current research have shown that IL‐23 inhibits trophoblast proliferation, migration and EMT via activating p38 MAPK signalling pathway.^18^ Hence, exploring the effects of miRNAs and the p38 MAPK signalling pathway is essential to study SA mechanisms.

Exosomes are membranous vesicles released outside the cell after the fusion of intracellular multivesicular body and cell membrane, with a diameter of about 30 to 150 nm.^19^ They exist in blood, urine, pleural effusion, cerebrospinal fluid, ascites, breast milk, saliva and other body fluids and participate in various physiological and pathological processes.^20^ Exosomes contain miRNAs,^21^, ^22^ mRNAs^21^, ^23^ and DNA sequences^24^, ^25^ that can be transferred to receptor cells to modulate their behaviour.^26^, ^27^, ^28^ Recently, increasing research regarding exosomes has found that exosomal miRNAs impact the regulation of embryonic implantation and endometrial epithelial cell secretion.^29^, ^30^ Thus, we hypothesized that serum exosome‐derived miRNAs from SA patients are related to SA occurrence. In‐depth studies of exosomes have shown that they are essential communication carriers for intercellular material transfer and signal transduction.^21^, ^31^, ^32^, ^33^ Engineered exosomes can transport long‐distance intercellular material and transmit signals for therapeutic effects.^21^, ^31^ Bovine milk is an essential source of exosomes since it is easy to obtain and harmless to dairy cows.^34^ Milk exosomes have several advantages, including being free of immune rejection and inflammatory responses. They are also scalable, biocompatible and inexpensive, which enhances their bioavailability and efficacy.^35^ Herein, we used milk exosomes as carriers to transfer eletrotrasnfected‐miRNA.

To test our hypothesis, systematic in vitro and in vivo experiments and clinical validation were performed. The results in vitro experiments showed that serum exosomes from SA patients suppressed trophoblast EMT, migration and invasion. By comparing serum exosomal miRNAs from normal control (NC) patients with abortion for unplanned pregnancy and SA patients, we identified miR‐410‐3p as the key miRNA that inhibited trophoblast EMT, migration and invasion. Mechanistically, SA serum exosomal miR‐410‐3p directly restrained the EMT by targeting TNF receptor‐associated factor 6 (TRAF6) to reduce trophoblast migration and invasion via the p38 MAPK signalling pathway. Furthermore, milk exosomes loaded with miR‐410‐3p mimic aggravated female mice's embryo absorption in vivo. Clinically, SA patients had abnormal miR‐410‐3p and TRAF6 expressions in placental villous tissues, and miR‐410‐3p was inversely correlated with TRAF6. These results elucidated novel mechanisms of serum exosomal miRNAs in SA and suggested that SA serum exosomal miR‐410‐3p exerts an important role in trophoblasts via the p38 MAPK signalling pathway.

## 2 MATERIALS AND METHODS

### 2.1 Patients, tissue and peripheral blood samples

Placental villous tissues and peripheral blood samples were obtained from 15 NC and 15 SA patients at the First Affiliated Hospital of Guangzhou University of Chinese Medicine (Guangzhou, China) between December 2020 and December 2021. The exclusion criteria were as follows: (a) other diseases such as endocrine and metabolic diseases; (b) abnormal uterine morphology; (c) husband and wife have chromosomal abnormalities; and (d) accepted other drug treatments before surgery. Samples were obtained with patient consent, and the procedures were approved by the Ethics and Internal Review Committee of First Affiliated Hospital of Guangzhou University of Chinese Medicine. Patient baseline characteristics are summarized in Table S1. Part of the tissues was fixed with 4% paraformaldehyde and blocked with paraffin. The rest was preserved at −80°C for exosome extraction and other experiments. Serum samples were obtained by centrifuging peripheral blood at 3000*g*/4°C for 15 min, then stored at −80°C.

### 2.2 Cell culture and reagents

The human trophoblast cell line HTR‐8/SVneo (HTR‐8) was purchased from the American Type Culture Collection (ATCC) and cultured at Lingnan Medical Research Center of Guangzhou University of Chinese Medicine. HTR‐8 cells grew in RPMI‐1640 medium (Gibco, USA) containing 10% fetal bovine serum (FBS) (Gibco) at 37°C and 5% CO_2_. Trypsin–EDTA (Gibco) was used to trypsinize cells for passaging and seeding.

### 2.3 Exosome isolation

Serum exosomes were isolated using cell culture media and the Umibio® Exosome Isolation Kit (Umibio, China) following the manufacturer's instructions. To remove cells and debris, an initial spin was performed at 3000 *g*/4°C for 10 min and then 10,000 *g*/4°C for 20 min. Next, the corresponding reagents were proportionally added to the starting sample volume. The mixtures were vortexed and incubated at 4°C for 2 h, then centrifuged at 10,000 *g*/4°C for 60 min to precipitate the exosome pellets. They were resuspended with 1× phosphate‐buffered saline (PBS) and purified with the exosome purification filter at 3000 *g*/4°C for 10 min.^36^, ^37^ Placental villous tissues were minced in PBS, and placental villous exosomes were isolated from the cell culture media, according to Leroyer et al.^38^ with minor modifications. After isolation, exosomes were stored at −80°C immediately for further analysis. Protein concentration was determined using the BCA Protein Assay Kit (Beyotime, China).

### 2.4 Transmission electron microscopy

Exosome morphology was determined by transmission electron microscopy (TEM). First, exosomes were resuspended in 50 to 100 μL 2% paraformaldehyde. 5 μL of this exosome suspension was added to formvar–carbon‐coated copper grids. PBS was added to the parafilm, and the copper grids were placed with the formvar membrane side down on the PBS droplet with tweezers to wash. The copper grids were placed on 50 μL 1% glutaraldehyde droplet for 5 min and washed eight times for 2 min in 100 μL double‐distilled water. Then, copper grids were placed on 50 μL uranyl oxalate droplets for 5 min and 50 μL methylcellulose droplets for 10 min on ice. Copper grids were placed on the stainless steel ring at the sample stage top; filter paper was used to absorb excess liquid and then dried in air for 5 min. Images were acquired from TEM (JEOL, Japan).

### 2.5 Nanoparticle tracking analysis

Exosome particle size and concentration were measured by nanoparticle tracking analysis (NTA) at VivaCellBiosceinces with ZetaView PMX 110 (Particle Metrix, Germany) and ZetaView 8.5 software. When necessary, isolated exosomes were diluted properly. NTA measurement was conducted at 11 positions. The ZetaView system was calibrated using 110 nm polystyrene particles. The temperature was maintained at 27.68°C.

### 2.6 Cellular internalization of exosomes

The NC and SA serum exosomes were labelled with PKH26 fluorescent dye (Umibio, China) following the manufacturer's instructions. Stained exosomes were co‐cultured with HTR‐8 cells. After 48 h of incubation, the nuclei of each group were stained with Antifade Mounting Medium with DAPI (Beyotime). The fluorescence was determined for each group using an IX71 fluorescence microscope (Olympus, Japan).

### 2.7 Cell transfection

The lentivirus for TRAF6 (LV TRAF6) and negative control (LV Ctrl) were purchased from GeneChem Technology (China). The miRNA mimic and inhibitor for miR‐410‐3p and the siRNA for TRAF6 were synthesized by RiboBio (USA). HTR‐8 cells were seeded on 6‐well plates 24 h before transfection. When cells reached 40%–50% confluence, mimic and inhibitor miRNAs, or siRNA, were transfected with Lipofectamine™ 3000 (Invitrogen, USA) following the manufacturer's instructions. Cells were harvested 48 h after transfection.

### 2.8 RNA isolation and quantitative real‐time PCR

Total RNA was isolated using MagZol Reagent (Magen, China). For mRNA expression analysis, cDNA was synthesized with RevertAid First Strand cDNA Synthesis Kit (ThermoFisher, USA), and RNA isolation and quantitative real‐time PCR (RT‐PCR) was performed using PowerUp™ SYBR™ Green Master Mix (ThermoFisher). For miRNA expression analysis, reverse transcription was performed with All‐in‐One™ miRNA First‐Strand cDNA Synthesis Kit 2.0 (GeneCopoeia, USA), and RT‐PCR was performed using All‐in‐One™ miRNA qPCR Kit (GeneCopoeia). GAPDH or U6 was used for standardization. Relative mRNA and miRNA levels were calculated using the 2^−ΔΔ*Ct*^ method. Primers were provided by Sangon Biotech (China) (Table S2).

### 2.9 Western blotting

The total protein of serum exosomes, placental villous exosomes, placental villous tissues and cells was isolated using the RIPA Lysis Buffer (Beyotime) and quantified by the BCA Protein Assay Kit. Samples were boiled at 100°C for 5 min in 5× sample loading buffer, separated on 8–15% SD‐polyacrylamide gel, and transferred to 0.22 or 0.45 μm PVDF membranes. Membranes were blocked with 5% skim milk for 2 h or QuickBlock™ Blocking Buffer (Beyotime) for 20 min at room temperature (RT) and incubated with primary antibodies overnight at 4°C. Then, samples were incubated with the appropriate secondary antibody labelled with horseradish peroxidase. Chemiluminescent signals were detected using Femto‐sig ECL Western Blotting Substrate (Tanon, China). Images were captured using a chemiluminescence imaging system (Bio‐Rad USA). Densitometric protein level analysis was performed using Image Lab. Primary antibodies against E‐cadherin (Cat# 20874‐1‐AP), N‐cadherin (Cat# 22018‐1‐AP), vimentin (Cat# 10366‐1‐AP), GAPDH (Cat# 10494‐1‐AP) and the secondary antibody Goat Anti‐Rabbit IgG (H + L) (Cat# SA00001‐2) were purchased from Proteintech (USA); primary antibodies against p38 MAPK (Cat# 8690 T) and Phospho‐p38 MAPK (Cat# 4511 T) from Cell Signaling Technology (USA); primary antibodies against CD6 (Cat# ab193349), CD81 (Cat# ab83) and TSG101 (Cat# ab109201) from Abcam (UK).

### 2.10 Cell Counting Kit‐8 (CCK‐8) assay

The CCK‐8 assay was used to assess cell proliferation ability. HTR‐8 cells were seeded in 96‐well plates and treated with corresponding interventions for different periods. Cell viability was detected by CCK‐8 (Dojindo Laboratories Japan). The absorbance at 450 nm was detected using a microplate reader (Therom, USA).

### 2.11 Scratch wound healing assay

The scratch wound healing assay was performed to assess cell migration ability. HTR‐8 cells were seeded in 6‐well plates and grown to full confluency. Cell monolayers were wounded by 200 μL pipette tip scratching, washed with PBS and incubated in serum‐free media. After 48 h, the images were acquired using the inverted microscope (Olympus). The wound healing rate was analysed by Image‐Pro Plus.

### 2.12 Transwell assay

The transwell assay was performed to assess cell invasion ability. HTR‐8 cells were seeded in 24‐well plates with Falcon® Permeable Support for 24‐well plates and an 8.0 μm Transparent PET Membrane (Corning, USA). HTR‐8 cells were added in 200 μL FBS‐free medium and placed in the upper chamber of each insert covered with Matrigel (Corning). The lower chamber was filled with 500 μL medium containing 10% FBS. The plate was incubated at 37°C for 48 h. Cells were then fixed in 4% paraformaldehyde at room temperature followed by permeabilization with 100% methanol for 5 min and crystal violet staining for 20 min. Images were analysed and quantified by Image J.

### 2.13 Dual‐luciferase reporter assay

Luciferase vectors containing miR‐410‐3p wild‐type and mutated binding sites in the 3′‐UTR of TRAF6 (mut‐3′‐UTR: from GGGCUUUAUGUUGUAU to CCGGAUAAACAAGAUA) were transfected into HTR‐8 cells using Lipofectamine™ 3000 (Invitrogen). After 24 h of transfection, the relative luciferase activity was measured using the TransDetect® Double‐Luciferase Reporter Assay Kit (TransGen Biotech, China).

### 2.14 Milk exosomes loading

The miR‐410‐3p mimic was delivered to milk exosome extracts (Umibio) as previously described.^39^, ^40^, ^41^ First, we prepared ECM 630 Electroporation System (BTX, USA) and Gene Pulser/MicroPulser Electroporation Cuvettes (RiboBio). Then, miR‐410‐3p mimic and its negative mimic control (mimic Ctrl) were centrifuged at 12,000 rpm/4°C for 2 min and dissolved in DEPC water (final concentration: 1 μg/μL). Next, they were added to milk exosomes and gently mixed to form Exo‐mimic Ctrl or Exo‐miR‐410‐3p mimic. Electroporation Cuvettes were added with the mixture to the motor slot with the following parameters: 400 V, 200 Ω, 125 uF. Immediately after one electric shock, the mixture was placed on ice for 5 min, then filtered with a 0.22 μm filter and stored at −80°C for later use.

### 2.15 Animal experiments

Eight‐week‐old female and male C57BL/6 and male BALB/c mice were obtained from OBiO Technology (Shanghai) Corp., Ltd. Female mice were divided into four groups: control (Ctrl): C57BL/6 females mated with C57BL/6 males (*n* = 4); model (Model): C57BL/6 females mated with BALB/c males (*n* = 4); model + mimic Ctrl‐loaded exosomes (Model + Exo‐mimic Ctrl): C57BL/6 females mated with BALB/c males, milk exosomes loaded with mimic Ctrl were administered to female mice via tail vein injections (*n* = 4); model + miR‐410‐3p mimic‐loaded exosomes (Model + Exo‐miR‐410‐3p mimic): C57BL/6 females mated with BALB/c males, milk exosomes loaded with miR‐410‐3p mimic were administered to female mice via tail vein injections (*n* = 4). The female‐to‐male mating ratio was 2:1. After determining the concentration gradient for the pre‐experiment, 180 μg milk exosomes loaded with mimic Ctrl or miR‐410‐3p mimic by electrotransfection or PBS was injected into female mice on plug detection day (Day 0). Injections were repeated every 3 days, and mice were sacrificed on Day 11. The embryo resorption rate was calculated, and miR‐410‐3p and TRAF6 levels were detected.

### 2.16 Imaging of fluorescently labelled exosomes and in vivo and ex vivo tracking

Milk exosomes loaded with mimic Ctrl or miR‐410‐3p mimic were labelled with the lipophilic, near‐infrared fluorescent dye 1,1‐dioctadecyl‐3,3,3,3‐tetramethylindotricarbocyaine iodide (DiR Iodide) (YEASEN, China). After injection, in vivo and ex vivo fluorescence images of different organs (brain, heart, lung, spleen, liver, kidney and embryo) were obtained. Fluorescence intensity was quantified using the IVIS® LUMINA SERIES III and Living Image® 4.5.2 software (PerkinElmer, USA) to assess tissue distribution of DiR Iodide‐labelled exosomes.

### 2.17 Immunohistochemistry

After washing with PBS, tissues were fixed in 10% formaldehyde for 4–6 h at RT, followed by routine deparaffinization and dehydration for Immunohistochemistry (IHC). Formalin‐fixed paraffin‐embedded tissues were cut into 4 μm sections. Endogenous peroxidase activity was blocked with 3% hydrogen peroxide and nonspecific binding with 5% bovine serum albumin for 20 min. Tissue sections were incubated with primary antibodies targeting TRAF6 for 1 h at 37°C and then washed three times with PBS plus 1% Tween‐20 (PBST) for 3 min. Antibody binding was detected with a brown precipitate followed by 3,3‐diaminobenzidine (DAB) staining (ZSGB BIO, China), haematoxylin counterstaining and dehydration. IHC slides were scanned by a Pannoramic DESK scanner and analysed by CaseViewer (3DHISTECH, Hungary).

### 2.18 In situ hybridization

Tissue sections were deparaffinized in xylene, hydrated in graded ethanol series and washed with DEPC water. Then, they were boiled in antigen retrieval solution for 10 min. Proteinase K was added, and samples were digested at 37°C for 15 min. After PBS washing, 3% methanol‐H_2_O_2_ was added to tissue sections and incubated at RT for 20 min. After PBS washing, the pre‐hybridization solution was added to tissue sections and incubated at 37°C for 1 h. The pre‐hybridization solution was removed, and the hybridization solution containing miR‐410‐3p probe with a concentration of 500 nM was added and then incubated at 40°C for overnight hybridization. Salt sodium citrate (SSC) was used to wash off the hybridization solution and block tissue sections at RT for 30 min. Then, the blocking solution was removed, and mouse anti‐digoxigenin‐labelled peroxidase (anti‐DIG‐HRP) was added for incubation at 4°C overnight. Results were visualized after colour development using DAB and haematoxylin counterstaining. Finally, sections were dehydrated in graded ethanol, cleared in xylene and mounted with coverslips. In situ hybridization (ISH) slides were scanned by a Pannoramic DESK scanner and analysed by CaseViewer.

### 2.19 Immunofluorescence staining

Tissue sections were treated with xylene and graded alcohol and heated with citric acid for antigen retrieval. After blocking with 10% serum for 1 h at RT, sections were incubated overnight with primary antibodies against trophoblast marker cytokeratin 7 (CK7) (Cat# 22018‐1‐AP) (Proteintech) and TRAF6 at 4°C. The following day, sections were incubated with fluorochrome‐conjugate rabbit and mouse secondary antibodies (Invitrogen) and mounted with Antifade Mounting Medium with DAPI. Observe sections with an IX71 fluorescence microscope.

## 3 RESULTS

### 3.1 SA serum exosomes suppress trophoblast EMT, proliferation, migration and invasion

**FIGURE 1:**
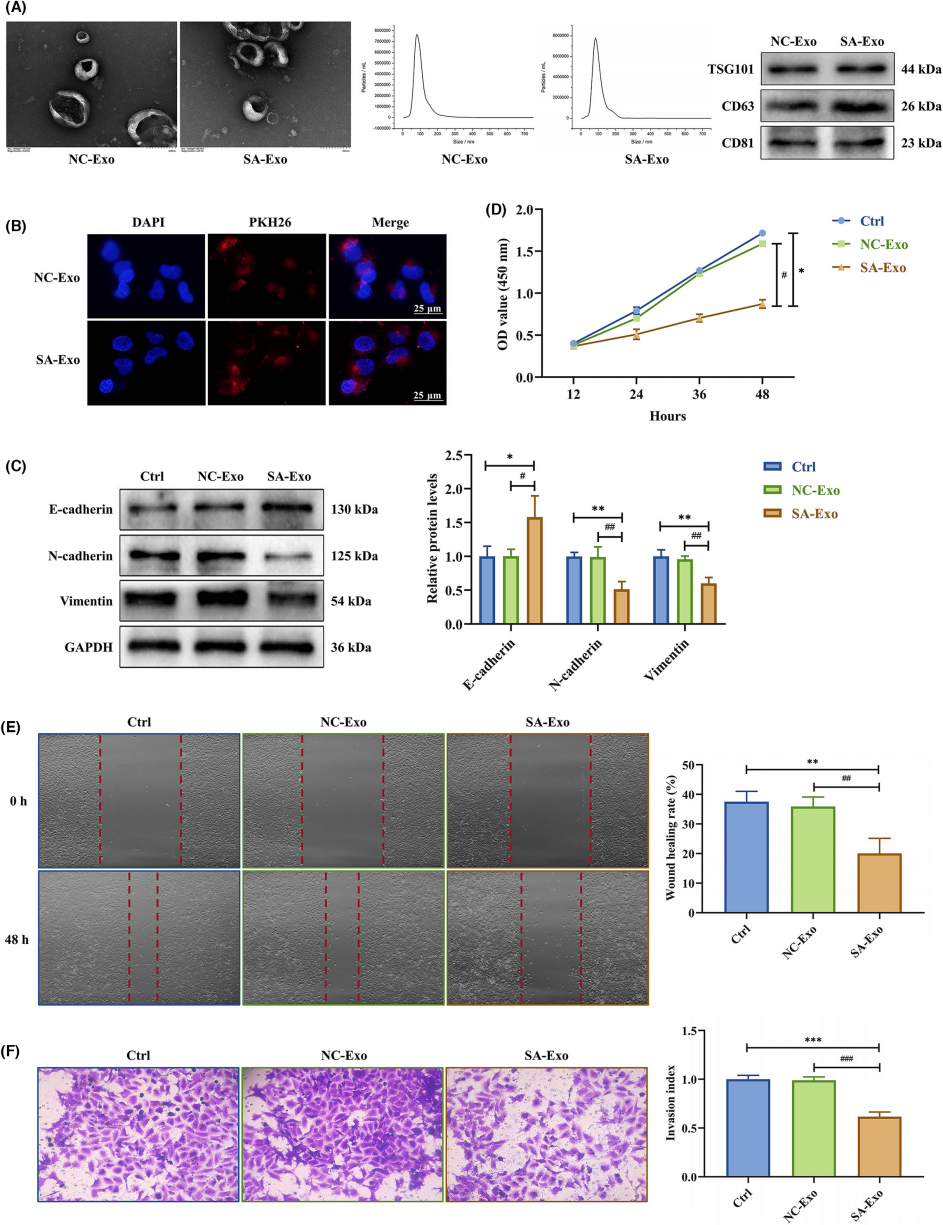
SA serum exosomes suppress EMT, proliferation, migration and invasion of trophoblasts. (A) The morphology of milk exosomes was observed by TEM (Scale bar, 200 nm), and the diameter distribution of milk exosomes was detected by NTA. Besides, the expression levels of milk exosomes specific markers were detected by western blotting. (B) NC or SA serum exosomes were internalized, respectively, by HTR‐8 cells. Blue colour represented nuclei stained with DAPI, and red colour represented exosomes stained with PKH26 (Scale bar, 25 μm). (C) The expression levels of E‐cadherin, N‐cadherin and vimentin protein in HTR‐8 cells treated with NC or SA serum exosomes were tested by western blotting. (D) Proliferation capacity in HTR‐8 cells treated with NC or SA serum exosomes was detected by CCK‐8 assay. (E,F) Migration and invasion capacities in HTR‐8 cells treated with NC or SA serum exosomes were measured by scratch wound healing and transwell assays, respectively. Representative images of migrated or invaded cells are shown (magnification, ×200). Error bars, SD. *p < 0.05, **p < 0.01, ***p < 0.001, ^#^ *p* < 0.05, ^##^ *p* < 0.01, ^###^ *p* < 0.001, vs indicated group.

Isolate exosomes from the serum of 15 NC and 15 SA patients. The TEM showed that exosomes displayed typical cup‐shaped morphology. The NTA revealed that the exosome diameter distribution was 30 to 150 nm. Besides, the western blot revealed that the specific markers CD81, CD63 and TSG101 were abundant in exosomes (Figure 1A). These results confirmed that the microvesicles were exosomes.

Immediately afterwards, we co‐cultured exosomes with HTR‐8 cells to observe whether they were taken up by HTR‐8 cells. PKH26‐labelled exosomes overlapped with DAPI‐labelled HTR‐8 cells, indicating that HTR‐8 cells successfully internalized exosomes (Figure 1B). To investigate whether SA serum exosomes regulated trophoblast EMT in vitro, we co‐cultured HTR‐8 cells with NC or SA serum exosomes, and EMT markers were detected by western blotting. SA serum exosomes upregulated E‐cadherin (epithelial marker) while downregulated N‐cadherin and vimentin (mesenchymal markers) in HTR‐8 cells (Figure 1C).

CCK‐8, wound healing and transwell assays were then performed to confirm whether SA serum exosomes influenced trophoblast proliferation, migration and invasion abilities. Compared to control and NC serum exosomes groups, HTR‐8 cells co‐cultured with SA serum exosomes displayed lower proliferative capacity (Figure 1D), slower scratch closure (Figure 1E) and lower invasion (Figure 1F). These results demonstrated that SA serum exosomes suppressed trophoblast EMT, proliferation, migration and invasion.

### 3.2 SA serum exosomes are enriched with miR‐410‐3p

Moreover, miRNAs are critical for many biological processes, including cell proliferation, differentiation and migration.^42^ The in‐depth study of miRNA functions in diseases has shown that aberrant miRNA expression is related to disease occurrence and onset, and changes in miRNA expression profiles are common in human diseases.^43^, ^44^ Several studies have shown that exosomal miRNAs might be associated with gamete development, embryogenesis and other related diseases.^45^, ^46^ Exosomal miRNAs might also be non‐invasive biomarkers to predict disease states.^47^, ^48^, ^49^

**FIGURE 2:**
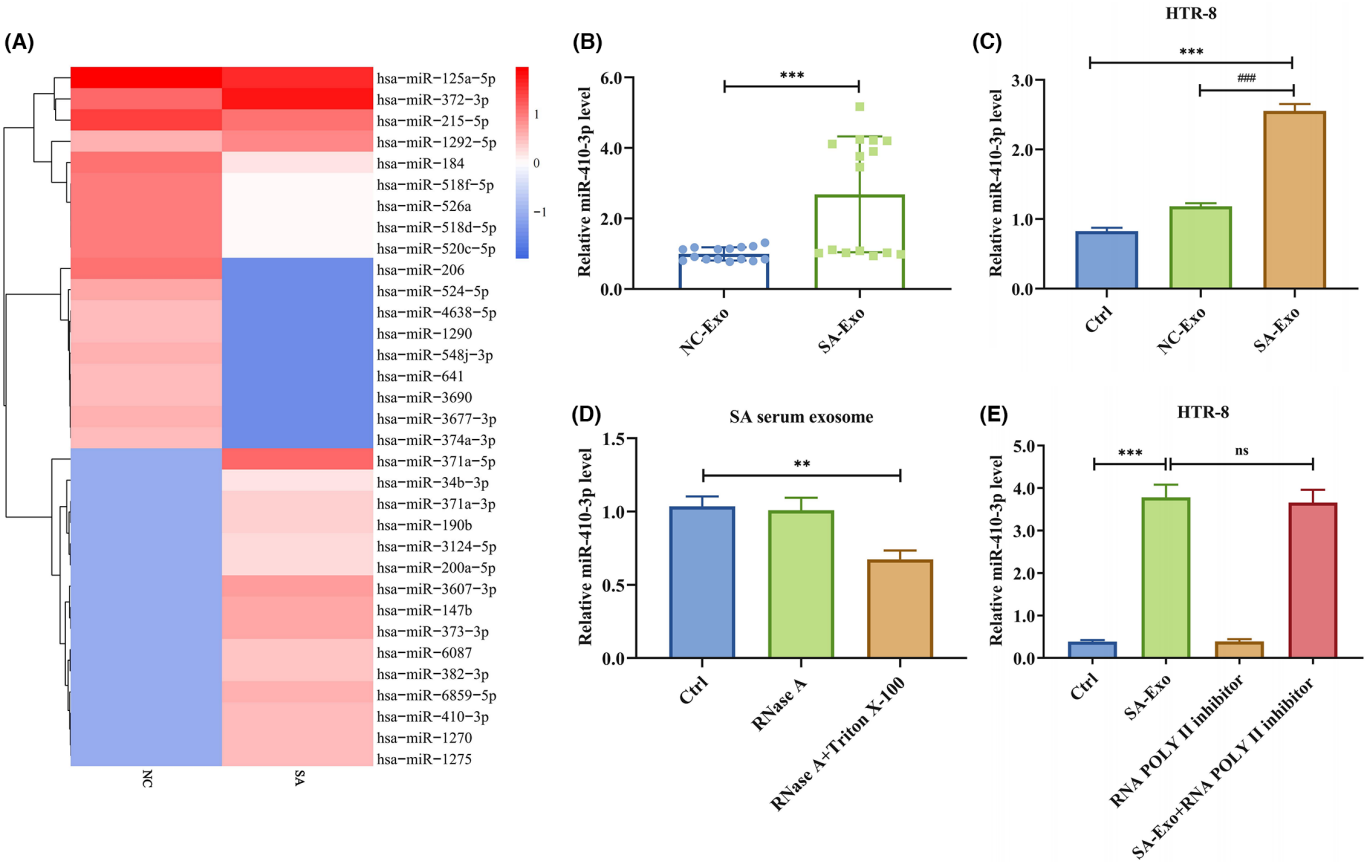
SA serum exosomes are enriched with miR‐410‐3p. (A) The heatmap showed the relative expression of miRNAs in NC and SA serum exosomes. (B) The expression level of miR‐410‐3p in NC and SA serum exosomes was measured by RT‐PCR. (C) The expression level of miR‐410‐3p in HTR‐8 cells treated with NC or SA serum exosomes was detected by RT‐PCR. (D) The expression level of miR‐410‐3p in SA serum exosomes treated with RNase A alone or in combination with Triton X‐100 was detected by RT‐PCR. (E) The expression level of miR‐410‐3p in HTR‐8 cells treated with polymerase II inhibitors and then incubated with SA serum exosomes was monitored by RT‐PCR. Error bars, SD. **p < 0.01, ***p < 0.001, ^###^ *p* < 0.001, n.s., not significant, vs indicated group.

Thus, we sequenced miRNAs from NC and SA serum exosomes to determine how they affected trophoblast EMT, migration and invasion. The heatmap showed that miR‐371a‐5p (log2(foldchange) = 8.0579, *p*‐value = 0.004751), miR‐3607‐3p (log2(foldchange) = 6.2209, *p*‐value = 0.010145), miR‐147b (log2(foldchange) = 5.9556, *p*‐value = 0.000715), miR‐373‐3p (log2(foldchange) = 5.9193, *p*‐value = 0.013759), miR‐6859‐5p (log2(foldchange) = 5.6017, *p*‐value = 0.004176), miR‐410‐3p (log2(foldchange) = 5.4236, *p*‐value = 0.009131), miR‐1275 (log2(foldchange) = 5.3395, *p*‐value = 0.039169), miR‐1270 (log2(foldchange) = 5.3295, *p*‐value = 0.005300), miR‐382‐3p (log2(foldchange) = 5.128, *p*‐value = 0.028083) and miR‐6087 (log2(foldchange) = 4.9745, *p*‐value = 0.012158) were the top 10 differentially expressed miRNAs (Figure 2A). Since only miR‐410‐3p is a human‐mouse homologous, we detected its expression in dependent samples by RT‐PCR. We demonstrated that miR‐410‐3p was highly expressed in SA serum exosomes (Figure 2B). Hence, we selected miR‐410‐3p as the pivotal miRNA to study.

Then, we treated HTR‐8 cells with NC or SA serum exosomes to elucidate whether the exosomes regulated SA serum inhibitory activity. The RT‐PCR showed that SA serum exosomes increased miR‐410‐3p levels (Figure 2C). The miR‐410‐3p level was reduced in SA serum exosomes after RNase A and Triton X‐100 treatment but not after RNase A treatment alone (Figure 2D), suggesting that extracellular miR‐410‐3p was encapsulated in the cell membrane rather than released directly. Furthermore, preliminary RNA polymerase II inhibitor treatment did not impact the level of miR‐410‐3p in recipient HTR‐8 cells treated with SA serum exosomes (Figure 2E), indicating that the increased miR‐410‐3p levels in trophoblasts might be due to SA serum exosomes‐mediated miRNA transfer rather than endogenous miR‐410‐3p induction. These findings revealed that SA serum exosomes are enriched with miR‐410‐3p and increase miR‐410‐3p levels in trophoblasts.

### 3.3 miR‐410‐3p is responsible for the effects of SA serum exosomes on trophoblasts

**FIGURE 3:**
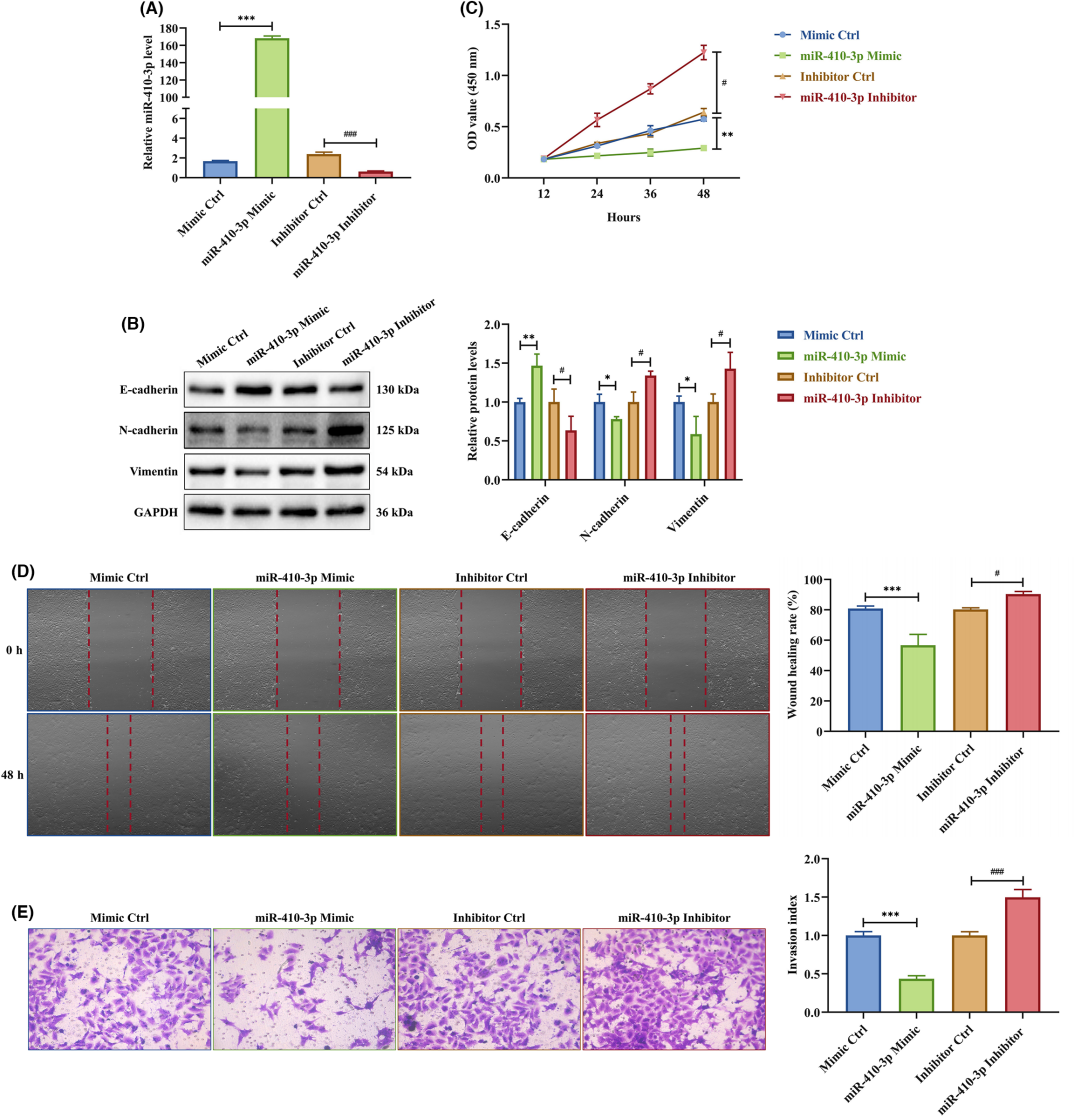
miR‐410‐3p is responsible for the effects of SA serum exosomes on trophoblasts. (A) The expression level of miR‐410‐3p in HTR‐8 cells transfected with miR‐410‐3p mimic or inhibitor was measured by RT‐PCR. (B) The expression levels of E‐cadherin, N‐cadherin and vimentin protein in HTR‐8 cells transfected with miR‐410‐3p mimic or inhibitor were detected by western blotting. (C) Proliferation capacity in HTR‐8 cells transfected with miR‐410‐3p mimic or inhibitor was detected by CCK‐8 assay. (D,E) Migration and invasion capacities in HTR‐8 cells transfected with miR‐410‐3p mimic or inhibitor were determined by scratch wound healing and transwell assays, respectively. Representative images of migrated or invaded cells are shown (magnification, ×200). Error bars, SD. *p < 0.05, **p < 0.01, ***p < 0.001, ^#^ *p* < 0.05, ^###^ *p* < 0.001, vs indicated group.

To study the role of miR‐410‐3p on trophoblast migration and invasion, HTR‐8 cells were transfected with miR‐410‐3p mimic or inhibitor. The miR‐410‐3p mimic facilitated miR‐410‐3p expression in HTR‐8 cells, the opposite of its inhibitor (Figure 3A). Additionally, the miR‐410‐3p mimic induced E‐cadherin and inhibited N‐cadherin and vimentin protein levels in HTR‐8 cells in contrast to the miR‐410‐3p inhibitor (Figure 3B). In the CCK‐8 assay, the miR‐410‐3p mimic significantly inhibited HTR‐8 cell proliferation, while the miR‐410‐3p inhibitor stimulated it (Figure 3C). The wound healing and transwell assays showed that the miR‐410‐3p mimic slowed the scratch closure (Figure 3D) and weakened the invasion (Figure 3E) of HTR‐8 cells, the opposite of the miR‐410‐3p inhibitor. These results indicated that SA serum exosomes restrict trophoblast proliferation, migration and invasion via miR‐410‐3p transferring.

### 3.4 TRAF6 is a target gene of SA serum exosome‐delivered miR‐410‐3p

**FIGURE 4:**
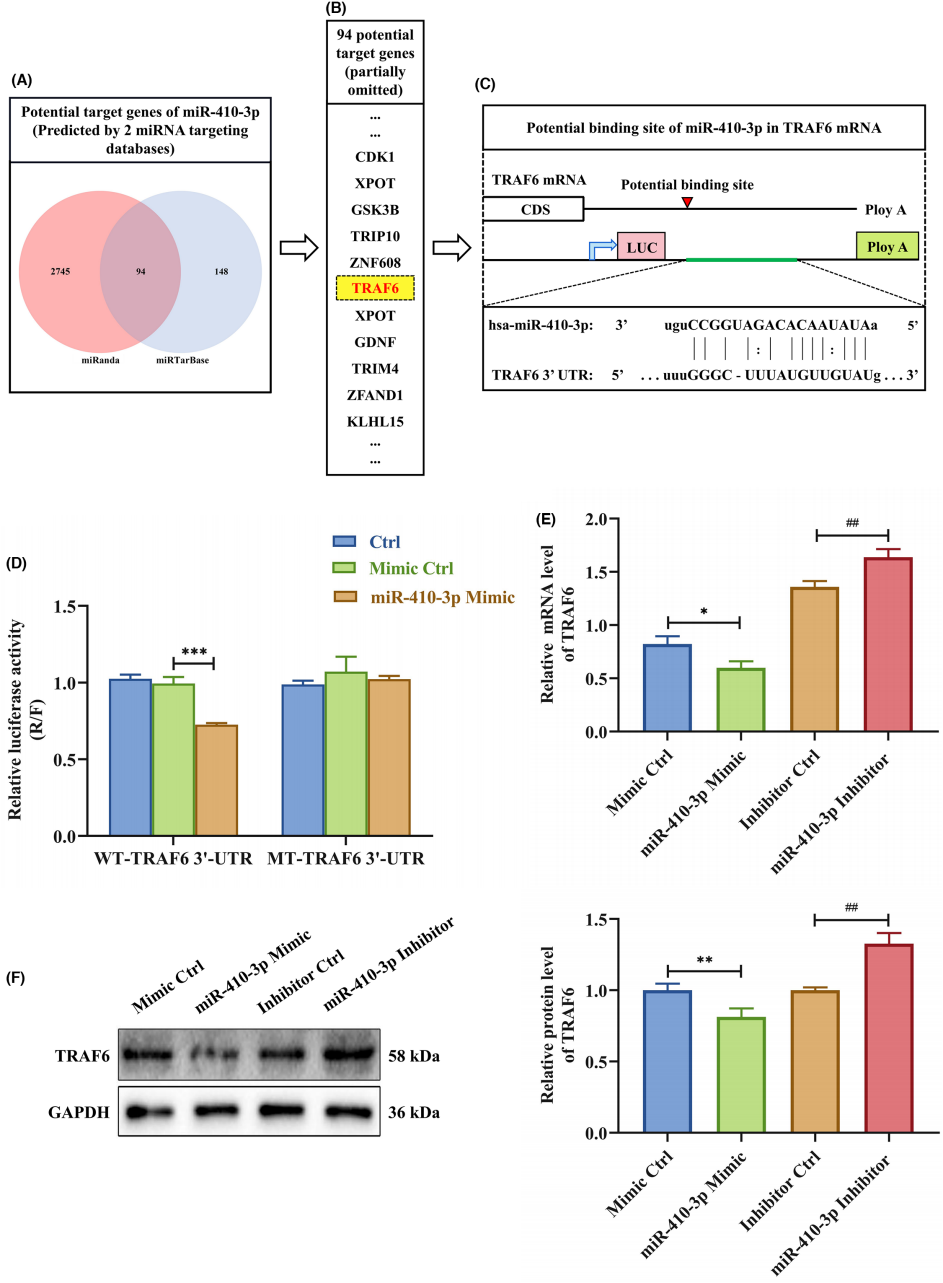
TRAF6 is a target gene of SA serum exosome‐delivered miR‐410‐3p. (A) Two independent miRNA target databases were used to predict potential target genes. (B) Potential target genes of miR‐410‐3p. (C) The schematic diagram of the TRAF6 3′‐UTR. Mutations were generated at the predicted miR‐410‐3p binding sites located in the TRAF6 3′‐UTR. (D) Dual‐luciferase reporter assay in HTR‐8 cells co‐transfected with luciferase reporter vector carrying wild‐type or mutant binding site of miR‐410‐3p in 3′‐UTR of TRAF6 as well as mimic Ctrl and miR‐410‐3p mimic. (E,F) The levels of TRAF6 mRNA and protein in HTR‐8 cells after transfection with miR‐410‐3p mimic or inhibitor were determined by RT‐PCR and western blotting, respectively. Error bars, SD. *p < 0.05, **p < 0.01, ***p < 0.001, ^##^ *p* < 0.01, vs indicated group.

Several researches have shown that miRNAs mainly exert the biological functions by adjusting target gene expression.^50^, ^51^ Thus, we used miRWalk, TargetScan, miRTarBase, miRDB and miRanda public databases to predict miR‐410‐3p target genes. Target genes were detected in miRTarBase and miRanda databases (Figure 4A). The potential miR‐410‐3p target genes were analysed, and TRAF6 was finally selected (Figure 4B).

Next, functional annotation clusters for these genes were analysed via literature review and David bioinformatic analysis. The online miRNA database prediction indicated that the 3′‐UTR of TRAF6 was a potential miR‐410‐3p binding site (Figure 4C). Hence, we co‐transfected luciferase vectors driven by wild‐type or mutant TRAF6 3′‐UTR miRNA binding sites with miR‐410‐3p mimics into HTR‐8 cells to identify whether TRAF6 is a direct miR‐410‐3p target. The miR‐410‐3p overexpression inhibited the luciferase activity of wild‐type TRAF6 3′‐UTR compared to the control group. Meanwhile, this inhibition was rescued by miR‐410‐3p binding site mutation (Figure 4D). To assess the impacts of miR‐410‐3p on TRAF6 expression, we transfected miR‐410‐3p mimic or inhibitor into HTR‐8 cells. TRAF6 protein and mRNA levels were decreased by miR‐410‐3p mimic and promoted by miR‐410‐3p inhibition (Figure 4E,F). These results clarify that TRAF6 is a target gene of miR‐410‐3p.

**FIGURE 5:**
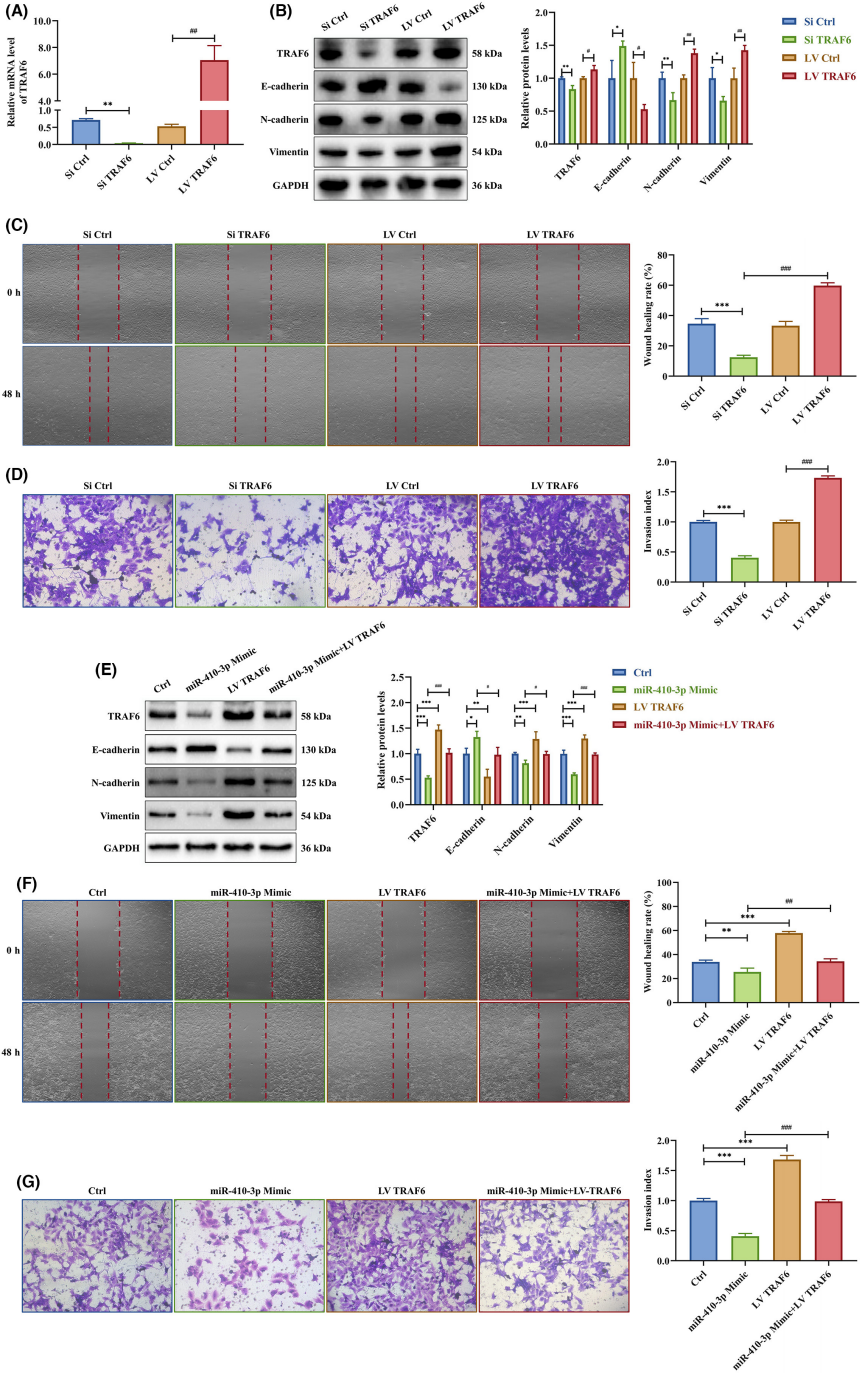
TRAF6 is a target gene of SA serum exosome‐delivered miR‐410‐3p. (A,B) The expression levels of TRAF6, E‐cadherin, N‐cadherin and vimentin mRNA and protein in HTR‐8 cells transfected with siTRAF6 or LV TRAF6 were measured by RT‐PCR and western blotting, respectively. (C,D) Migration and invasion capacities in HTR‐8 cells transfected with siTRAF6 or LV TRAF6 were determined by wound healing and transwell assays, respectively. Representative images of migrated or invaded cells are shown (magnification, ×200). (E) The expression levels of TRAF6, E‐cadherin, N‐cadherin and vimentin protein in HTR‐8 cells transfected with miR‐410‐3p mimic, LV TRAF6 alone or a combination of the two intervention were measured by western blotting. (F,G) Migration and invasion capacities in HTR‐8 cells transfected with miR‐410‐3p mimic, LV TRAF6 alone or a combination of the two intervention were determined by wound healing and transwell assays, respectively. Representative images of migrated or invaded cells are shown (magnification, ×200). Error bars, SD. *p < 0.05, **p < 0.01, ***p < 0.001, ^#^ *p* < 0.05, ^##^ *p* < 0.01, ^###^ *p* < 0.001, vs indicated group.

Next, we transfected HTR‐8 cells with siTRAF6 or LV TRAF6 to investigate the effects of TRAF6 on trophoblasts. Western blotting and RT‐PCR demonstrated that siTRAF6 downregulated and LV TRAF6 upregulated the expression of TRAF6 in HTR‐8 cells. Besides, siTRAF6 upregulated E‐cadherin and downregulated N‐cadherin and vimentin protein levels. The LV TRAF6 intervention had the opposite effects (Figure 5A,B). siTRAF6 decreased HTR‐8 cell migration and invasion abilities significantly, while LV TRAF6 promoted them (Figure 5C,D). Furthermore, LV TRAF6 rescued miR‐410‐3p mimic inhibition on TRAF6, N‐cadherin and vimentin protein levels and suppressed the E‐cadherin elevation promoted by miR‐410‐3p mimic in HTR‐8 cells (Figure 5E). LV TRAF6 rescued the repression of HTR‐8 cell migration and invasion abilities by miR‐410‐3p mimic transfection (Figure 5F,G). These results showed that miR‐410‐3p suppresses trophoblast EMT, migration and invasion via TRAF6 downregulation.

**FIGURE 6:**
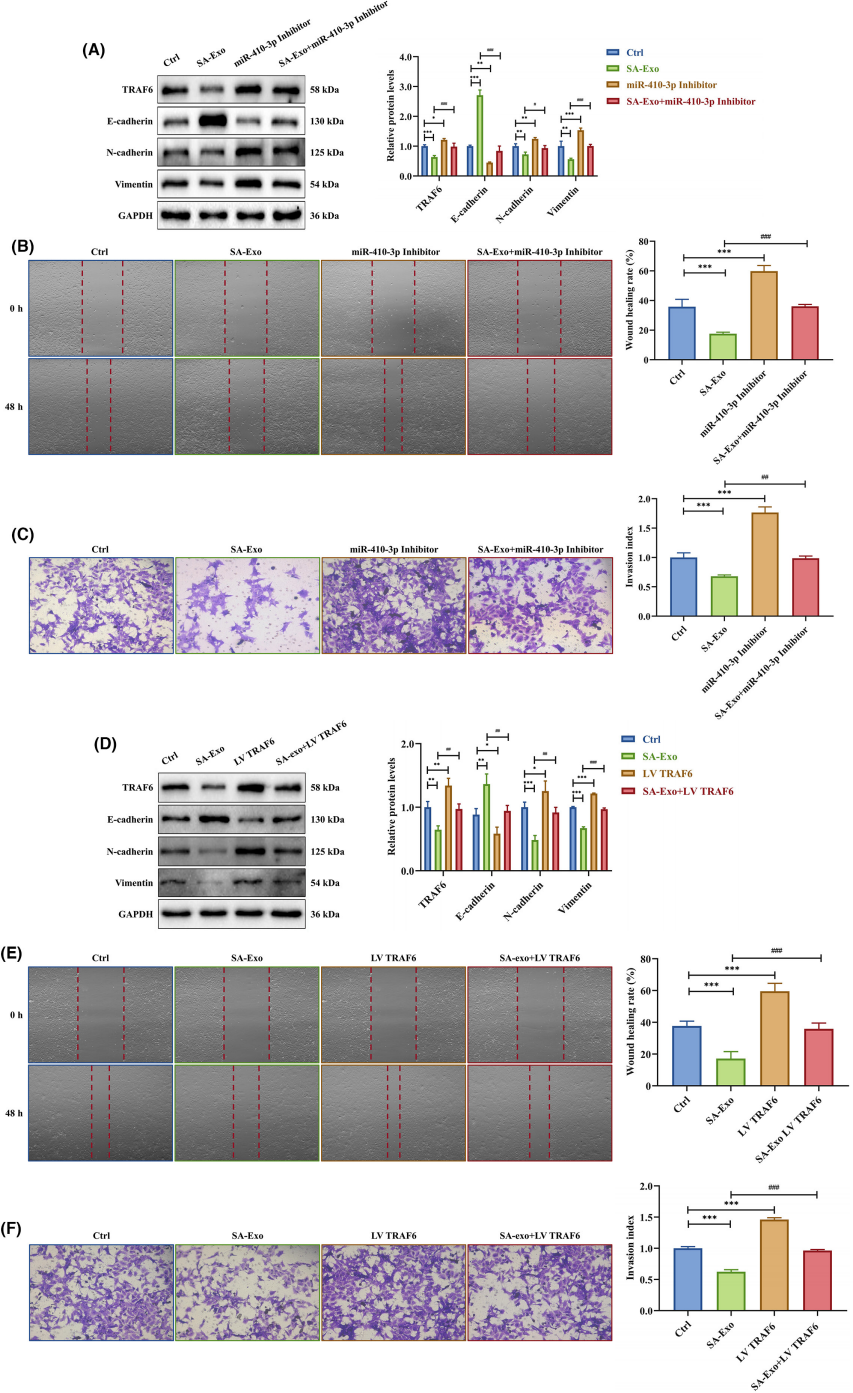
TRAF6 is a target gene of SA serum exosome‐delivered miR‐410‐3p. (A) The expression levels of TRAF6, E‐cadherin, N‐cadherin and vimentin protein in HTR‐8 cells co‐cultured with SA serum exosomes, transfected with miR‐410‐3p inhibitor, or intervened together were measured by western blotting. (B,C) Migration and invasion capacities in HTR‐8 cells co‐cultured with SA serum exosomes, transfected with miR‐410‐3p inhibitor or intervened together were determined by wound healing and transwell assays, respectively. Representative images of migrated or invaded cells are shown (magnification, ×200). (D) The expression levels of TRAF6, E‐cadherin, N‐cadherin and vimentin protein in HTR‐8 cells co‐cultured with SA serum exosomes, transfected with LV TRAF6 or intervened together were measured by western blotting. (E,F) Migration and invasion capacities in HTR‐8 cells co‐cultured with SA serum exosomes, transfected with LV TRAF6 or intervened together were determined by wound healing and transwell assays, respectively. Representative images of migrated or invaded cells are shown (magnification, ×200). Error bars, SD. *p < 0.05, **p < 0.01, ***p < 0.001, ^#^ *p* < 0.05, ^##^ *p* < 0.01, ^###^ *p* < 0.001, vs indicated group.

Further, HTR‐8 cells were co‐cultured with SA serum exosomes, transfected with miR‐410‐3p inhibitor or both. The western blot indicated that the effects of SA serum exosomes on TRAF6, N‐cadherin, vimentin downregulation and E‐cadherin upregulation were prevented by miR‐410‐3p inhibitor transfection into HTR‐8 cells (Figure 6A). The transfection with miR‐410‐3p inhibitor also restored the inhibitory effects of SA serum exosomes on HTR‐8 cell migration and invasion (Figure 6B,C).

Similarly, HTR‐8 cells were co‐cultured with SA serum exosomes, transfected with LV TRAF6 or both. The western blotting identified that TRAF6, N‐cadherin, vimentin downregulation and E‐cadherin upregulation induced by SA serum exosomes were prevented by LV TRAF6 transfection into HTR‐8 cells (Figure 6D). Besides, LV TRAF6 transfection rescued the SA serum exosome inhibition on HTR‐8 cell migration and invasion abilities (Figure 6E,F). These results suggested that SA serum exosome‐delivered miR‐410‐3p suppresses trophoblast EMT, migration and invasion via TRAF6 downregulation.

Altogether, these results demonstrated that TRAF6 is a target gene of SA serum exosome‐delivered miR‐410‐3p.

### 3.5 TRAF6‐mediated p38 MAPK signalling pathway is dampened by SA serum exosome‐delivered miR‐410‐3p

**FIGURE 7:**
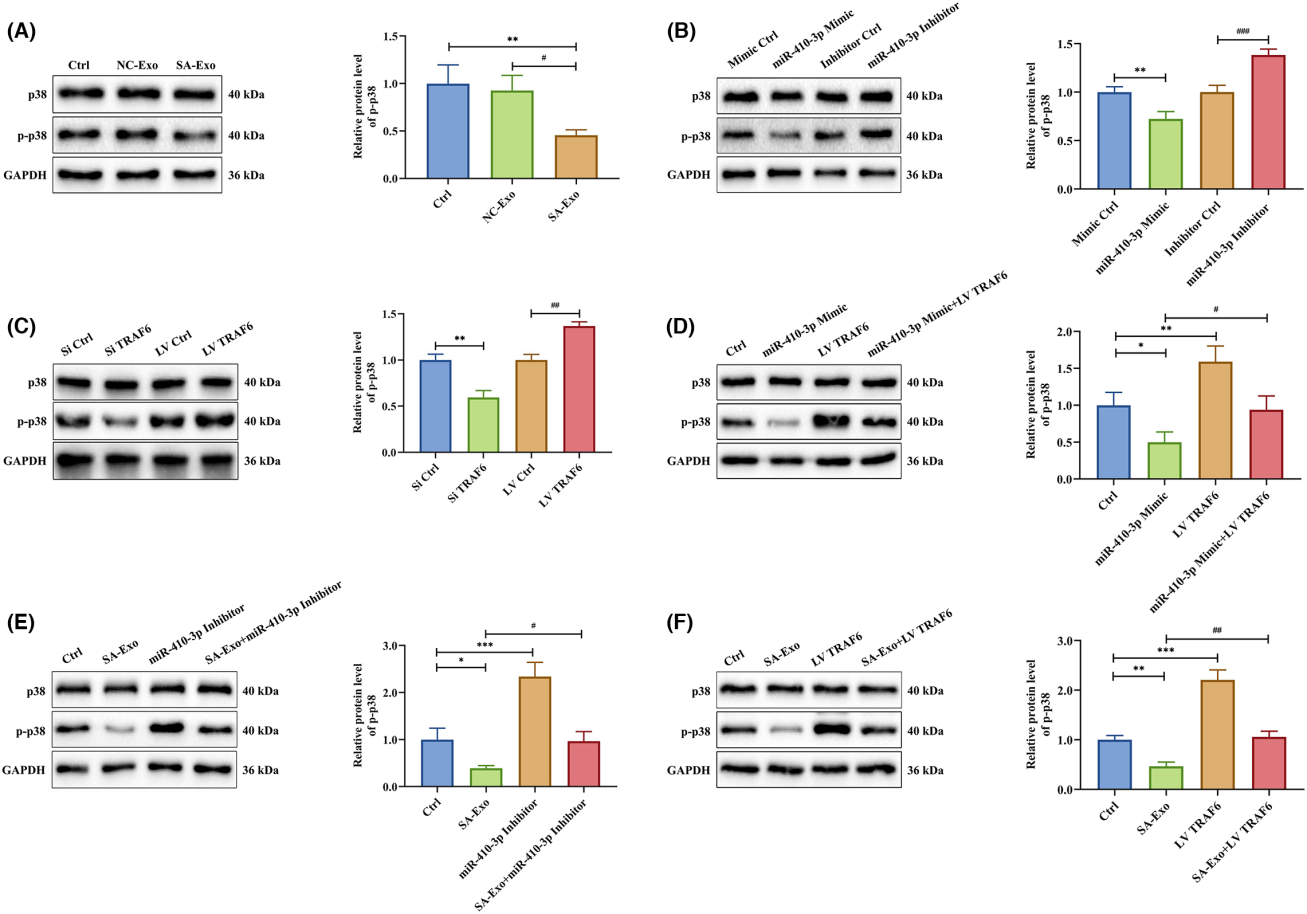
TRAF6‐emdiated p38 MAPK signalling pathway is dampened by SA serum exosome‐delivered miR‐410‐3p. (A) The expression levels of p38 and p‐p38 protein in HTR‐8 cells treated with NC or SA serum exosomes were detected by western blotting. (B) The expression levels of p38 and p‐p38 protein in HTR‐8 cells transfected with miR‐410‐3p mimic or inhibitor were measured by western blotting. (C) The expression levels of p38 and p‐p38 protein in HTR‐8 cells transfected with siTRAF6 or LV TRAF6 were determined by western blotting. (D) The expression levels of p38 and p‐p38 protein in HTR‐8 cells transfected with miR‐410‐3p mimic, LV TRAF6 alone or a combination of the two intervention were tested by western blotting. (E) The expression levels of p38 and p‐p38 protein in HTR‐8 cells co‐cultured with SA serum exosomes, transfected with miR‐410‐3p inhibitor or intervened together were detected by western blotting. (F) The expression levels of p38 and p‐p38 protein in HTR‐8 cells co‐cultured with SA serum exosomes, transfected with LV TRAF6 or intervened together were measured by western blotting. Error bars, SD. *p < 0.05, **p < 0.01, ***p < 0.001, ^#^ *p* < 0.05, ^##^ *p* < 0.01, ^###^ *p* < 0.001, vs indicated group.

The miR‐410‐3p target gene, TRAF6, is upstream of the p38 MAPK pathway. Hence, exploring the p38 MAPK signalling pathway mechanisms is crucial. To determine whether the p38 MAPK signalling pathway was affected by exosomal miR‐410‐3p, we co‐cultured HTR‐8 cells with NC or SA serum exosomes. The p‐p38 protein level decreased in HTR‐8 cells co‐cultured with SA serum exosomes compared to control and NC serum exosome groups (Figure 7A). When the miR‐410‐3p mimic or inhibitor was transfected into HTR‐8 cells, the miR‐410‐3p mimic inhibited p‐p38 expression in HTR‐8 cells, the opposite of its inhibitor (Figure 7B). After transfection of HTR‐8 cells with siTRAF6 or LV TRAF6, siTRAF6 downregulated and LV TRAF6 upregulated the expression p‐p38 (Figure 7C). Next, HTR‐8 cells were transfected with LV TRAF6, miR‐410‐3p mimic or both. The p‐p38 protein level was upregulated by LV TRAF6 and downregulated by miR‐410‐3p mimic. Meanwhile, LV TRAF6 rescued the miR‐410‐3p mimic inhibition on p‐p38 protein levels (Figure 7D). When HTR‐8 cells were co‐cultured with SA serum exosomes, transfected with miR‐410‐3p inhibitor or both, the effects of SA serum exosomes on p‐p38 downregulation were prevented by the miR‐410‐3p inhibitor (Figure 7E). Moreover, when HTR‐8 cells were co‐cultured with SA serum exosomes, transfected with LV TRAF6 or both, the effects of SA serum exosomes on p‐p38 downregulation were prevented by LV TRAF6 transfection (Figure 7F).

**FIGURE 8:**
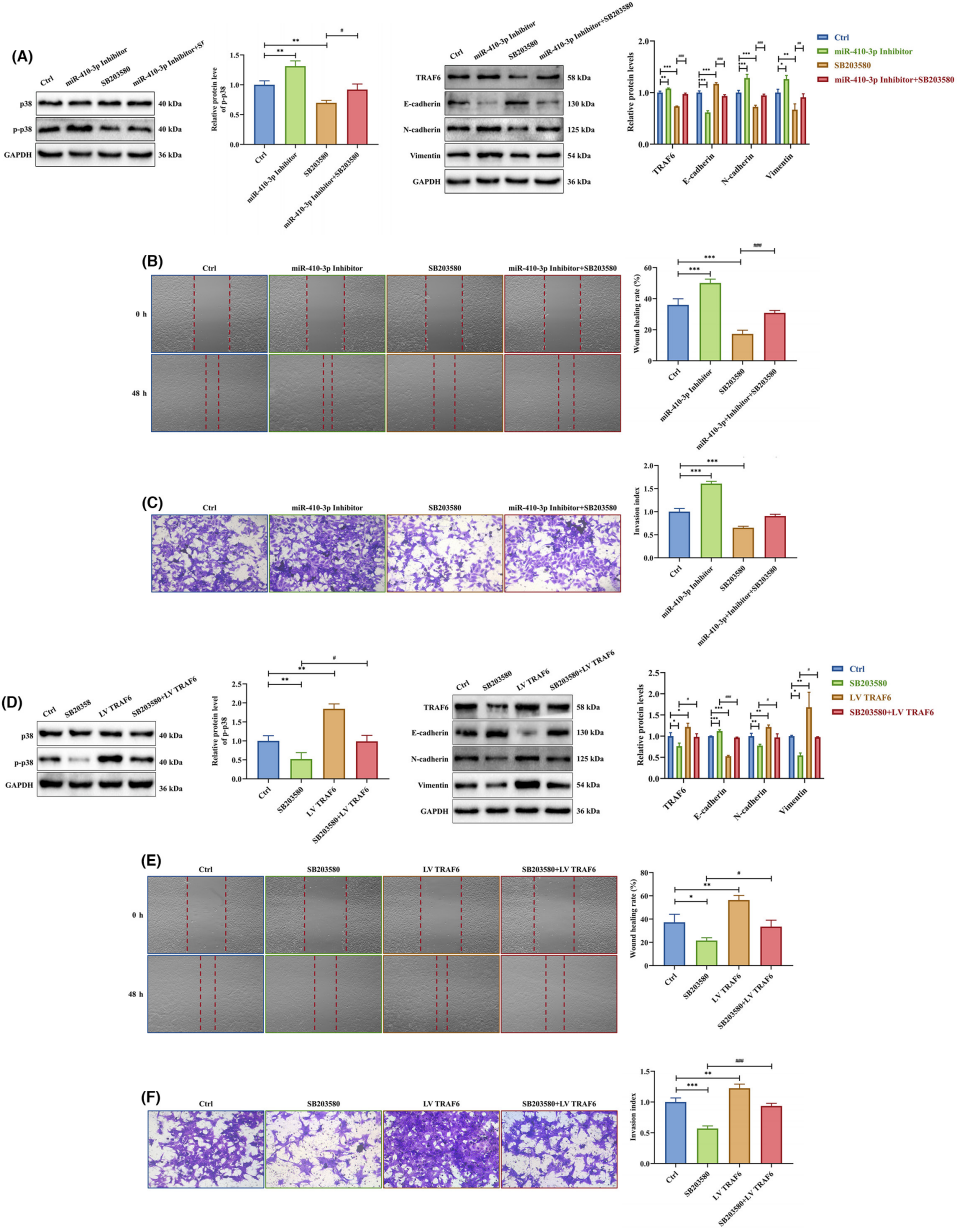
TRAF6‐emdiated p38 MAPK signalling pathway is dampened by SA serum exosome‐delivered miR‐410‐3p. (A) The expression levels of p38, p‐p38, TRAF6, E‐cadherin, N‐cadherin and vimentin protein in HTR‐8 cells cultured with SB203580, transfected with miR‐410‐3p inhibitor, or under the collective intervention were determined by western blotting. (B,C) Migration and invasion capacities in HTR‐8 cells cultured with SB203580, transfected with miR‐410‐3p inhibitor or intervened together were tested by wound healing and transwell assays, respectively. Representative images of migrated or invaded cells are shown (magnification, ×200). (D) The expression levels of p38, p‐p38, TRAF6, E‐cadherin, N‐cadherin and vimentin protein in HTR‐8 cells cultured with SB203580, transfected with LV TRAF6, or under the collective intervention were detected by western blotting. (E,F) Migration and invasion capacities in HTR‐8 cells cultured with SB203580, transfected with LV TRAF6 or intervened together were measured by wound healing and transwell assays, respectively. Representative images of migrated or invaded cells are shown (magnification, ×200). Error bars, SD. *p < 0.05, **p < 0.01, ***p < 0.001, ^#^ *p* < 0.05, ^##^ *p* < 0.01, ^###^ *p* < 0.001, vs indicated group.

To further investigate the p38 MAPK signalling pathway's important role in trophoblast migration and invasion, HTR‐8 cells were treated with the p38 MAPK signalling inhibitor SB203580 (MedChemExpress, USA) (24.11 μM). SB203580 inhibited p‐p38, TRAF6, N‐cadherin, and vimentin and promoted E‐cadherin expression, whereas miR‐410‐3p inhibitor reversed these effects in HTR‐8 cells (Figure 8A). Additionally, the miR‐410‐3p inhibitor rescued SB203580 inhibition on HTR‐8 cell migration and invasion abilities (Figure 8B,C). Similarly, LV TRAF6 reversed the inhibitory effects of SB203580 on p‐p38, TRAF6, N‐cadherin and vimentin protein levels and the stimulating effects on the E‐cadherin protein level in HTR‐8 cells (Figure 8D). Also, LV TRAF6 rescued SB203580 inhibition on HTR‐8 cell migration and invasion abilities (Figure 8E,F). These results showed that the TRAF6‐mediated p38 MAPK signalling pathway is attenuated by SA serum exosome‐delivered miR‐410‐3p in trophoblasts.

### 3.6 Milk exosomes loaded with miR‐410‐3p mimic promote embryo abortion

**FIGURE 9:**
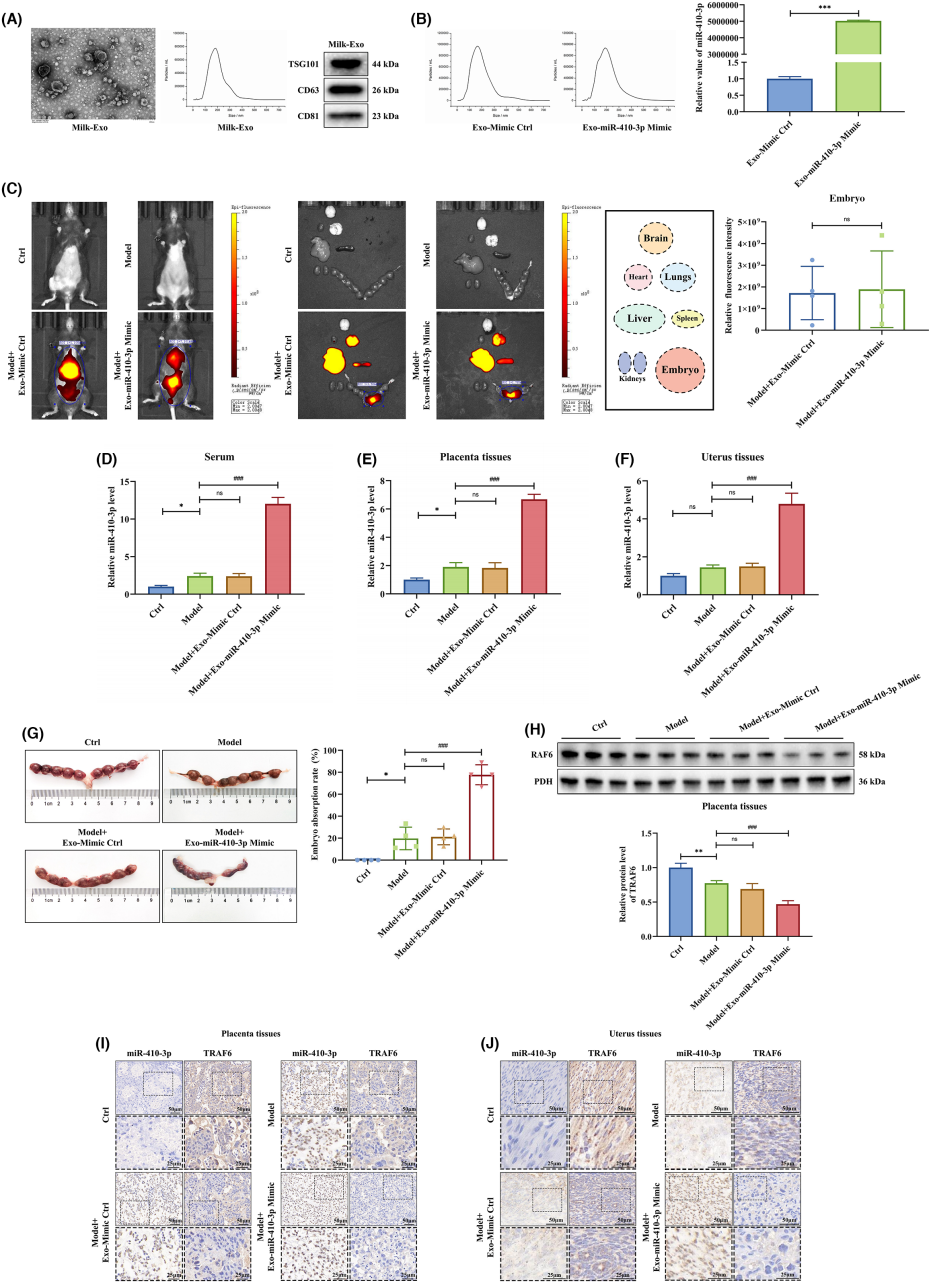
Milk exosomes loaded with miR‐410‐3p mimic promote embryo abortion. (A) The morphology of milk exosomes was observed by TEM (Scale bar, 200 nm), and the diameter distribution of milk exosomes was detected by NTA. Besides, the expression levels of milk exosomes specific markers were detected by western blotting. (B) The diameter distribution of milk exosomes loaded with mimic Ctrl or miR‐410‐3p mimic was detected by NTA, and the expression level of miR‐410‐3p in them was detected by RT‐PCR. (C) The in vivo fluorescence images of DiR Iodide‐labelled milk exosomes loaded with mimic Ctrl or miR‐410‐3p mimic in female mice (*n* = 4), the representative ex vivo fluorescence images in different organs (*n* = 4) and the quantification of ex vivo fluorescence images in embryos (*n* = 4) were detected. (D–F) The expression level of miR‐410‐3p in the serum, placenta and uterus of female mice were detected by RT‐PCR. (G) The embryo resorption rate of female mice of each group was calculated. (H) The expression level of TRAF6 protein in the placenta tissues of female mice was measured by western blotting. (I,J) The distributions of miR‐410‐3p and TRAF6 in the placenta and uterus of female mice of each group were detected by ISH and IHC, respectively (Scale bars, 50 μm, 25 μm). Error bars, SD. *p < 0.05, **p < 0.01, ***p < 0.001, ^###^ *p* < 0.001, n.s., not significant, vs indicated group.

To demonstrate the role of exosomal miR‐410‐3p in vivo, mimic Ctrl or miR‐410‐3p mimic were loaded into milk exosomes by electrotransfection. Milk exosomes were identified before electrotransfection by TEM and presented typical cup‐shaped morphology. The NTA showed that the diameter distribution of milk exosomes was 30 to 150 nm. The western blotting demonstrated that the specific markers CD81, CD63 and TSG101 were abundant in milk exosomes (Figure 9A). After electrotransfection, milk exosomes loaded with mimic Ctrl or miR‐410‐3p mimic were also detected by NTA and PCR. The diameter distribution of milk exosomes was still 30 to 150 nm, and miR‐410‐3p was highly expressed in them (Figure 9B). Then, we determined the fate of exosomes in vivo to clarify the role of milk exosomes loaded with miR‐410‐3p mimic in intercellular targeting and communication.

We explored the biodistribution of milk exosomes loaded with mimic Ctrl or miR‐410‐3p mimic in the circulation of C57BL/6 female mice mated with BALB/c male mice. The epifluorescence images of exosomes loaded with mimic Ctrl or miR‐410‐3p mimic were visualized in female mice. Besides accumulating in the lungs, livers and spleens, the fluorescence quantification revealed a significant accumulation of milk exosomes loaded with mimic Ctrl or miR‐410‐3p mimic in female mice embryos, indicating that they accessed the maternal‐fetal interface (Figure 9C). Next, miR‐410‐3p levels in serum, placenta and uterus were examined by RT‐PCR. This result showed that miR‐410‐3p was overexpressed in female mice injected with milk exosomes loaded with miR‐410‐3p mimic, including the serum, placenta and uterus (Figure 9D–F). Injection of milk exosomes loaded with miR‐410‐3p mimic also increased female mice embryo resorption significantly (Figure 9G). In addition, the abortion rates in mice of group Ctrl, Model, Model + Exo‐mimic Ctrl and Model + Exo‐miR‐410‐3p mimic were 0%, 25%, 25% and 100% respectively.

Furthermore, TRAF6 protein levels reduced in the placenta tissues of female mice injected with milk exosomes loaded with miR‐410‐3p mimic (Figure 9H). Then, we assessed the miR‐410‐3p and TRAF6 distribution in the placenta and uterine tissues by ISH and IHC. The ISH showed that miR‐410‐3p was located in the placenta tissues nucleus and the uterine tissues cytoplasm. The IHC indicated that TRAF6 was expressed in the cell membrane and cytoplasm of placenta tissues and the cytoplasm of uterine tissues. Notably, TRAF6 expression reduced in the placenta and uterine tissues of female mice injected with milk exosomes loaded with miR‐410‐3p mimic, the opposite of miR‐410‐3p expression (Figure 9I,J). These results showed that milk exosomes loaded with miR‐410‐3p mimic promoted embryo abortion by transferring miR‐410‐3p to suppress TRAF6 expression.

### 3.7 miR‐410‐3p is upregulated in placental villous exosomes and tissues from SA patients

**FIGURE 10:**
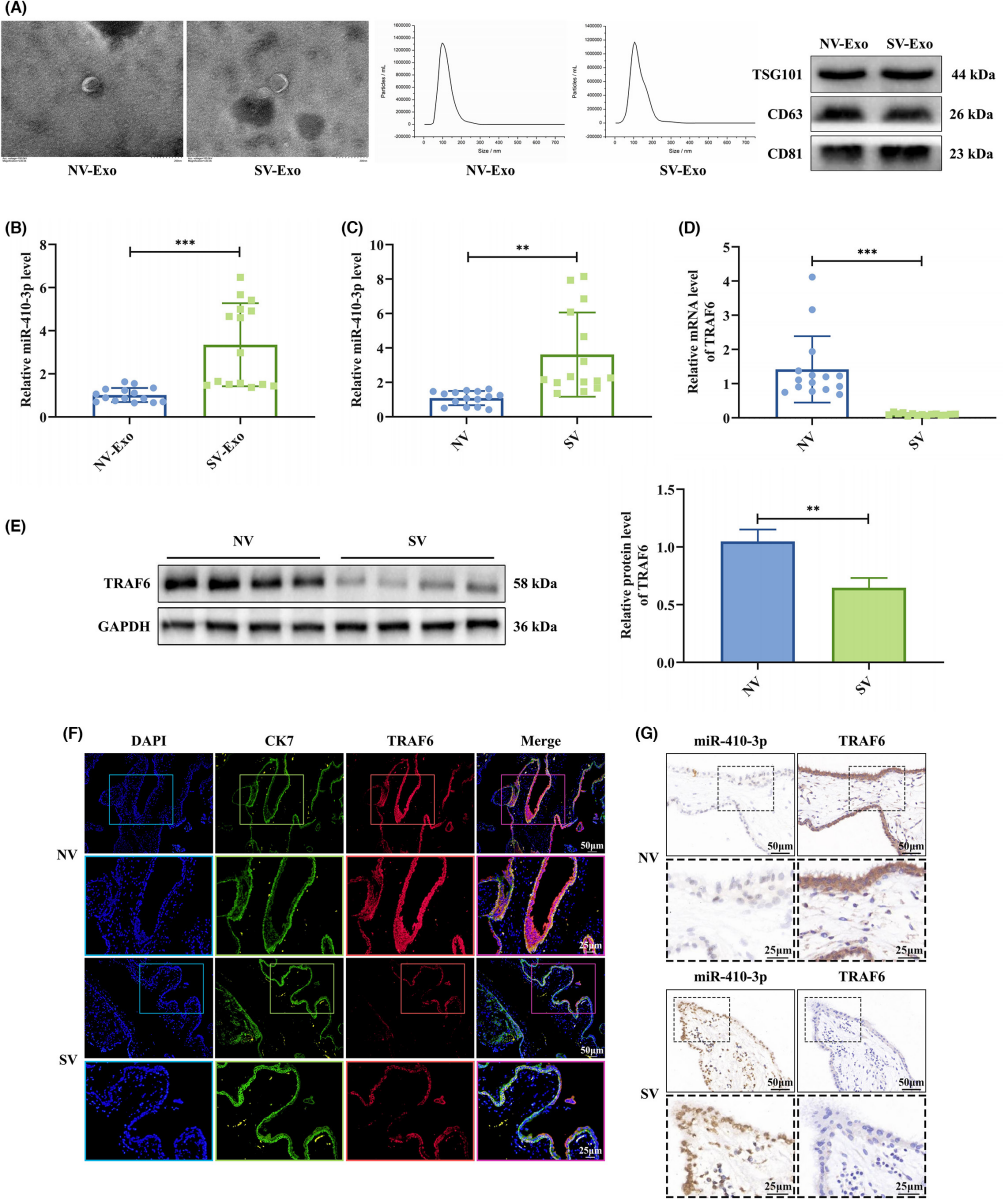
miR‐410‐3p is upregulated in placental villous exosomes and tissues from SA patients. (A) The morphology of placental villous exosomes was observed by TEM (Scale bar, 200 nm), and the diameter distribution of placental villous exosomes was detected by NTA. Besides, the expression levels of placental villous exosomes specific markers were detected by western blotting. (B) The expression level of miR‐410‐3p in the placental villous exosomes of NC patients (*n* = 15) and SA patients (*n* = 15) was measured by RT‐PCR. (C,D) The expression levels of miR‐410‐3p and TRAF6 mRNA in the placental villous tissues of NC patients (*n* = 15) and SA patients (*n* = 15) were measured by RT‐PCR. (E) The expression level of TRAF6 protein in the placental villous tissues of NC and SA patients was measured by western blotting. (F) Double IF staining was performed for trophoblast cell marker CK7 (green), TRAF6 (red) and nuclei DAPI (blue) in the placental villous tissues of NC and SA patients (Scale bar, 50 μm, 25 μm). (G) The distributions of miR‐410‐3p and TRAF6 in the placental villous tissues of NC and SA patients were detected by ISH and IHC, respectively (Scale bars, 50 μm, 25 μm). Error bars, SD. **p < 0.01, ***p < 0.001, vs indicated group.

Furthermore, NC and SA patients' placental villous tissues (NV, SV) were collected, and their exosomes were extracted and identified. The exosomes presented typical cup‐shaped morphology by TEM and diameter distribution of 30 to 150 nm by NTA. The western blotting demonstrated that the specific markers CD81, CD63 and TSG101 were abundant in placental villous exosomes (Figure 10A). Then, we investigated miR‐410‐3p and TRAF6 expression in placental villous exosomes and tissues from 15 NC and 15 SA patients. We found that miR‐410‐3p was highly expressed in placental villous exosomes of SA patients, which might be related to serum exosomes (Figure 10B). The elevated miR‐410‐3p level was also observed in placental villous tissues of SA patients (Figure 10C), combined with decreased TRAF6 mRNA and protein levels (Figure 10D,E). Double immunofluorescence (IF) staining of CK7 and TRAF6 further confirmed the TRAF6 reduction in placental villous tissues of SA patients (Figure 10F). Also, ISH and IHC showed that miR‐410‐3p and TRAF6 were mainly located in the nucleus, and miR‐410‐3p expression was negatively correlated with TRAF6 (Figure 10G). These clinical data demonstrated that miR‐410‐3p is significantly involved in SA development and progression.

## 4 DISCUSSION

Herein, we showed that SA serum exosomes restrained trophoblast EMT, migration and invasion by transferring miR‐410‐3p to target TRAF6, suggesting that miR‐410‐3p‐enriched SA serum exosomes might lead to SA pathogenesis. As far as we know, this is the first research to investigate the effects of SA serum exosome miRNAs on trophoblasts and their role in SA.

Several studies have explored the role of defective trophoblast migration and invasion in SA pathogenesis.^10^, ^48^, ^52^, ^53^, ^54^ The EMT, first discovered during embryonic development, occurs during trophoblast invasion as part of its differentiation process.^55^, ^56^, ^57^, ^58^, ^59^ Besides, EMT deficiency in placental trophoblasts affects postoperative and pregnancy complications.^60^, ^61^, ^62^, ^63^, ^64^, ^65^, ^66^ In the EMT, epithelial cells transform into mesenchymal cells via various complex molecular events, signalling pathways, epigenetic regulation and other mechanisms,^57^, ^67^, ^68^ exerting a vital role in regulating EVT migration and invasion.^12^, ^69^, ^70^ Since then, it has been related to embryonic development and fibrosis, tumour cell invasion and metastasis.^71^ Under the influence of EMT‐inducing signals, epithelial cells attenuate cell–cell adhesion, restrict the expression of epithelial cell markers and activate mesenchymal genes. All the changes confer on epithelial cells the ability to migrate and invade.^72^

Exosomes are vesicle‐like structures secreted by various cells. They are important carriers and act as an information exchange media between cells.^73^, ^74^, ^75^ Exosomes also act as messengers between the foetus and mother. Meanwhile, exosomes released by the mother support the growth and survival of the foetus. Exosome concentration is significantly higher in the peripheral blood of pregnant women than in non‐pregnant women.^76^, ^77^ In healthy pregnant women, plasma concentrations of placental exosomes increase as pregnancy progresses, peaking at the end of pregnancy.^78^, ^79^

Plantz et al.^80^ demonstrated the presence of exosomes in bovine skim milk in 1973, which have been proven biologically active^81^ and recognized as a potential exosome source.^34^ Exosomes are useful as vehicles of drug delivery for various reasons, including biocompatibility across species, longer circulating half‐life, uptake by other cells, and hydrophilic and lipophilic source of macromolecular load. Exosomes are also able to cross the blood–brain barrier^82^, ^83^, ^84^, ^85^ and the placental barrier.^86^, ^87^ Milk exosomes and associated miRNAs can survive conditions highly detrimental to nucleic acids^88^, ^89^, ^90^ due to their stability in acidic environments,^91^, ^92^ offering additional advantages as ideal oral drug carriers with broad therapeutic applications. Moreover, the uptake and effective delivery of various small molecules using milk exosomes have been demonstrated.^93^ For example, Radha Munagala et al.^34^ showed milk exosomes' versatility, carrying capacity and ability to target cancer. This was the first report to identify a low‐cost biocompatible exosome drug that improves oral bioavailability, efficacy and safety. Moreover, Farrukh Aqil et al.^93^ used milk exosomes as a novel system for siRNA delivery and demonstrated that they could deliver endogenous RNAs into recipient cells. In this present study, we used milk exosomes as a carrier of electrotransfected miR‐410‐3p mimic.

The 3′‐UTR of over 60% of human protein‐coding genes might be miRNA targets, potentially regulating health and disease conditions.^94^, ^95^ Since exosomes are rich in miRNAs, one of the most abundant biologically active substances,^21^, ^22^ exosomal miRNA profiling can provide information on gene expression patterns and recipient cell data on producer cell states.^96^ Increasing evidence has suggested that exosomal miRNAs regulate homeostasis and disturbances during pregnancy.^97^, ^98^, ^99^ Ying et al.^100^ have reported that exosomes from URSA deciduous macrophages deliver miR‐153‐3p to trophoblasts to target the IDO gene and activate the STAT3 signalling pathway, inhibiting cell proliferation and migration and promoting URSA emergence. Ding et al.^48^ have found that M1‐Mφ‐derived exosomes directly inhibite TRAF6 at the post‐transcriptional level by transporting miR‐146a‐5p and miR‐146b‐5p, thereby reducing trophoblast EMT, migration and invasion, involved in the pathogenesis of RSA. Herein, we showed that SA serum exosomes transported miR‐410‐3p to trophoblasts, and miR‐410‐3p overexpression inhibited trophoblast migration and invasion in vitro, negatively affecting trophoblast EMT. Moreover, the miR‐410‐3p source in SA serum exosomes might be related to placental villous exosomes.

TRAF family proteins can activate mitogen‐activated protein kinase, B/glycogen synthase kinase 3β and other signalling pathways and participate in cell proliferation, differentiation, survival, apoptosis, immune and inflammatory responses, and other processes.^101^, ^102^, ^103^, ^104^ TRAF6, a classic TRAF member, is an adaptor protein that mediates extensive protein–protein interactions via its TRAF and RING finger domains with unusual E3 ubiquitin ligase activity.^105^ TRAF6 is of vital importance in pregnancy‐induced epithelial cell proliferation,^106^ and its downregulation reduces invasion and metastasis in melanoma and lung cancer.^107^, ^108^ Jin et al.^109^ have reported that TRAF6 is a direct miR‐3935 target, negatively affecting trophoblast EMT by inhibiting RGS2, which suggests that the miR‐3935/TRAF6/RGS2 axis might determine PE molecular pathogenesis.

Mitogen‐activated protein kinases (MAPKs) are crucial signalling components translating extracellular stimuli into broad cellular responses.^110^ They are members of distinct signalling cascades and serve as focal points in response to various extracellular stimuli.^111^ MAPKs can be divided into three main families in mammals: extracellular‐signal‐regulated kinases (ERKs), Jun amino‐terminal kinases (JNKs) and stress‐activated protein kinases (p38/SAPKs).^112^ Activation of p38 brings about inflammation, apoptosis, cell differentiation and cycle regulation.^113^, ^114^ A research have suggested that S100P plays a physiological role in the normal development of pregnancy by regulating the p38 MAPK signalling pathway, which might also regulate the proliferation of trophoblasts.^115^ Furthermore, HTR‐8 cell stimulation with corticotropin‐releasing hormone (CRH) results in transient p38 MAPK phosphorylation, blocked by astressin and the p38 MAPK inhibitor SB203580.^116^ Herein, we observed that the p38 MAPK signalling pathway changed under the action of SA serum exosomal miR‐410‐3p targeting TRAF6 and the p38 MAPK signalling pathway inhibitor SB203580.

## 5 CONCLUSIONS

**FIGURE 11:**
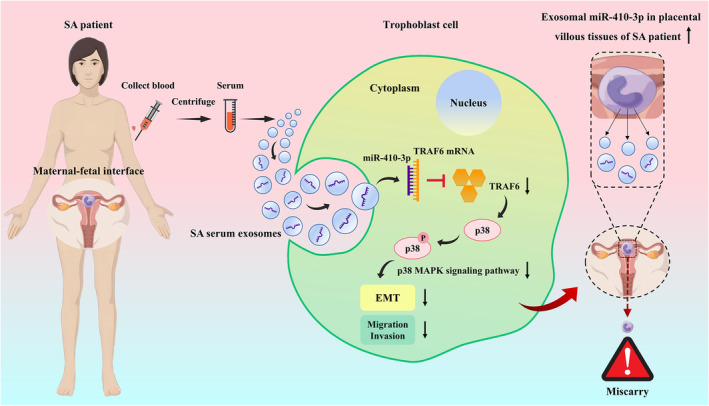
Schematic illustration of serum exosome‐derived miR‐410‐3p inhibits trophoblast EMT, migration and invasion in SA. SA serum exosomes could inhibit gestational trophoblast EMT, migration and invasion by transporting miR‐410‐3p to target TRAF6 through the p38 MAPK signalling pathway, thereby participating in the pathogenesis of SA.

In summary, we demonstrated that SA serum exosomes transport miR‐410‐3p to target TRAF6 via the p38 MAPK signalling pathway, thereby inhibiting gestational trophoblast EMT, migration and invasion of gestational trophoblasts, and participating in SA pathogenesis (Figure 11). These findings provide insights into the relationship between trophoblasts and serum exosomes in the maternal‐fetal interface microenvironment, representing a novel serum exosome regulation mechanism in SA. These findings emphasize that SA serum exosomal miR‐410‐3p is a promising therapeutic target for SA.

## AUTHOR CONTRIBUTIONS

**Gao‐pi Deng:** Conceptualization (lead); funding acquisition (lead); resources (lead). **Zhen‐yue Chen:** Conceptualization (lead); data curation (lead); writing – original draft (equal); writing – review and editing (equal). **Zhen Li:** Data curation (lead); writing – original draft (equal); writing – review and editing (equal). **Yun Zong:** Writing – original draft (supporting); writing – review and editing (supporting). **Bo Xia:** Writing – original draft (supporting); writing – review and editing (supporting). **Song‐ping Luo:** Funding acquisition (lead); resources (lead). **Jie Gao:** Conceptualization (lead); funding acquisition (lead); resources (lead).

## FUNDING INFORMATION

This work was supported by grants from the National Natural Science Foundation of China (81774358 and 82274565), the Key R&D Program of Guangdong Province (2020B1111100003), the ‘Double First‐Class’ and High‐level University Discipline Collaborative Innovation Team Project of Guangzhou University of Chinese Medicine (2021XK04), the Project of Department of Education of Guangdong Province (2021ZDZX2033) and City University (Institute) Joint Funding Project Guangzhou Key Laboratory Construction Project (202201020383).

## CONFLICT OF INTEREST STATEMENT

The authors declare that there is no conflict of interest.

## Supporting information


Table S1

## Data Availability

The data used to support the findings of the present study are available from the corresponding author upon request.
