# Supplementary material for: Exosome‐delivered miR‐410‐3p reverses epithelial‐mesenchymal transition, migration and invasion of trophoblasts in spontaneous abortion
Source: J Cell Mol Med. 2024 Jan 2;28(3):e18097. doi: 10.1111/jcmm.18097 (PMC10844701; doi:10.1111/jcmm.18097)
Supplement: Supplementary file 1 — Table S1 [file JCMM-28-e18097-s001.docx]

**Table S1 Comparison of the Baseline Data between NC and SA Group**

| Parameter | NC（n=15） | SA（n=15） | *p* |
| --- | --- | --- | --- |
| Age (years) | 27.333±1.878 | 27.867±1.767 | 0.883 |
| Gestational week | 6.000（6.000, 7.000） | 6.500（6.000, 7.000） | 0.389 |
| The number of pregnancy | 1.000（1.000, 2.000） | 1.000（1.000, 2.000） | 0.775 |
| The number of [parturition](javascript:;) | 0.000（0.000, 1.000） | 0.000（0.000, 1.000） | 1.000 |
| The number of SA | 0.000（0.000, 0.000） | 0.000（0.000, 0.000） | 0.775 |

Notes: The sample of each group was less than 50, so the Shapiro-Wilk test was used to evaluate the normal distribution. The age conforms to the normal distribution and is represented by（$\bar{x}\pm s$）, and two independent samples T test is used for comparison. The gestational week, the number of pregnancy, the number of [parturition](javascript:;), and the number of SA do not meet the normal distribution, so the Mann–Whitney U test was used for comparison, represented by *M* (*P25, P75*).

**Table S2. The Sequences of Primers for RT-PCR**

| Gene | Primer Sequence |
| --- | --- |
| hsa-miR-410-3p | 5' -ccgcgcgAATATAACACAGATGGCCTG- 3' |
| U6 | F: 5' -GCTTCGGCAGCACATATACTAAAAT- 3' |
|  | R: 5' -CGCTTCACGAATTTGCGTGTCAT- 3' |
| TRAF6 | F: 5' -GCACGCCACCTACAAGAGAACAC- 3' |
|  | R: 5' -CCAGAGTCGGGTATAACGCTCAAAC- 3' |
| GAPDH | F: 5' -ACCCAGAAGACTGTGGATGG- 3' |
|  | R: 5' -GAGGCAGGGATGATGTTCTG- 3' |
